# A *Pseudomonas aeruginosa* Toxin that Hijacks the Host Ubiquitin Proteolytic System

**DOI:** 10.1371/journal.ppat.1001325

**Published:** 2011-03-24

**Authors:** Jennifer M. Bomberger, Siying Ye, Daniel P. MacEachran, Katja Koeppen, Roxanna L. Barnaby, George A. O'Toole, Bruce A. Stanton

**Affiliations:** 1 Microbiology and Immunology, Dartmouth Medical School, Hanover, New Hampshire, United States of America; 2 Biology, Massachusetts Institute of Technology, Cambridge, Massachusetts, United States of America; Mt. Sinai Toronto, Canada

## Abstract

*Pseudomonas aeruginosa (P. aeruginosa)* is an opportunistic pathogen chronically infecting the lungs of patients with chronic obstructive pulmonary disease (COPD), pneumonia, cystic fibrosis (CF), and bronchiectasis. Cif (PA2934), a bacterial toxin secreted in outer membrane vesicles (OMV) by *P. aeruginosa*, reduces CFTR-mediated chloride secretion by human airway epithelial cells, a key driving force for mucociliary clearance. The aim of this study was to investigate the mechanism whereby Cif reduces CFTR-mediated chloride secretion. Cif redirected endocytosed CFTR from recycling endosomes to lysosomes by stabilizing an inhibitory effect of G3BP1 on the deubiquitinating enzyme (DUB), USP10, thereby reducing USP10-mediated deubiquitination of CFTR and increasing the degradation of CFTR in lysosomes. This is the first example of a bacterial toxin that regulates the activity of a host DUB. These data suggest that the ability of *P. aeruginosa* to chronically infect the lungs of patients with COPD, pneumonia, CF, and bronchiectasis is due in part to the secretion of OMV containing Cif, which inhibits CFTR-mediated chloride secretion and thereby reduces the mucociliary clearance of pathogens.

## Author Summary

In this manuscript, we present a detailed mechanistic study of how a secreted *P*. *aeruginosa* virulence factor disrupts mucociliary clearance in the lung by inactivating a host cell deubiquitinating enzyme (USP10), thereby facilitating the degradation of the CFTR secretory chloride channel, which reduces mucociliary clearance by human airway epithelial cells. To our knowledge, this is the first report of a bacterial toxin that alters host innate immune defense by hijacking host proteins involved in the ubiquitin proteolytic system. *P. aeruginosa* is recognized as the most common bacterial pathogen in ventilator-associated pneumonia and is commonly isolated from community-acquired pneumonia patients. While *P. aeruginosa* can cause devastating acute infections, many studies have documented that *P. aeruginosa* can also generate chronic infections in immunocompromised individuals, including cystic fibrosis, chronic obstructive pulmonary disease and bronchiectasis patients. In these patients, the ineffective host immune response to the bacterial colonization is thought to play a large role in deteriorating lung function and ultimately the death of the patient. Our findings have significant implications to the study of *P. aeruginosa* infections in the lung, particularly the ability of *P. aeruginosa* to disrupt critical host innate immune response mechanisms.

## Introduction

Respiratory infections are the greatest cause of disease worldwide [1]. A study by the World Health Organization (WHO) determined that the global disease burden of lung infections exceeds that of HIV/AIDS, cancer and heart disease and has since 1990 [1]. *P. aeruginosa*, an opportunistic human pathogen is commonly associated with respiratory infections, particularly nosocomial, ventilator-associated infections and pseudomonal pneumonia in immunocompromised patients, including cystic fibrosis, chronic obstructive pulmonary disease (COPD), ventilator-associated pneumonia, community-acquired pneumonia, and bronchiectasis patients. We have previously demonstrated that outer membrane vesicles (OMV) secreted by *P. aeruginosa* deliver multiple virulence factors into host human airway epithelial cells via a mechanism involving OMV fusion with airway cell plasma membrane lipid rafts and trafficking via an N-WASP induced actin pathway to deliver OMV cargo directly to the host cytoplasm [2]. This provides a mechanism for *P. aeruginosa* to alter host cell biology without the need for contact with airway epithelial cells, an important consideration in respiratory diseases where *P. aeruginosa* resides primarily in the mucus layer above the host airway epithelium [2].

Cif, a virulence factor secreted in OMV by clinical isolates of *P. aeruginosa,* was first described for its ability to decrease the apical membrane expression of the cystic fibrosis transmembrane conductance regulator (CFTR) and to reduce chloride secretion [3], [4], [5]. The Cif-induced reduction in the apical membrane abundance of CFTR in airway epithelial cells is due to an inhibition of the recycling of endocytic vesicles containing CFTR back to the plasma membrane and redirection of these vesicles to the lysosome where CFTR is degraded. The mechanism by which Cif reduces the recycling of endocytic vesicles containing CFTR is currently unknown, and thus, characterizing this mechanism was the goal of the present study.

Many bacteria-derived effectors regulate host pathways, including intracellular vesicular trafficking and ubiquitination, which targets proteins for degradation in the lysosome and proteasome [6], [7]. Pathogens frequently target the ubiquitination/deubiquitination systems of host cells to suppress the innate immune response and enhance pathogen colonization [8], [9]. For example, *Salmonella* produces a DUB, SslE, which reduces the cytotoxicity of *Salmonella* in macrophages [6]. Altered ubiquitin signaling involves the delivery by the pathogen of a DUB into host cells, thereby reducing the host response to bacterial pathogens.

In a recent study, we demonstrated that USP10, a host cell DUB, deubiquitinates CFTR in endosomes, thereby reducing the lysosomal degradation of CFTR, and maintaining cell and plasma membrane CFTR [10]. However, the effect of Cif on USP10 has not been examined. Thus, the goal of this study was to test the hypothesis that Cif inhibits USP10, which increases the amount of ubiquitinated CFTR that is degraded in lysosomes, thereby reducing cell and plasma membrane CFTR level. The data in this report demonstrates that Cif stabilizes an inhibitory effect of Ras-GAP SH3 domain binding protein-1 (G3BP1) on USP10, thereby reducing USP10 mediated deubiquitination of CFTR and increasing the degradation of CFTR in lysosomes. This is the first example of a bacterial toxin that regulates a host DUB. We propose that the ability of *P. aeruginosa* to chronically infect the lungs of patients with CF, pneumonia, COPD, and bronchiectasis is due in part to the secretion of OMV containing Cif, which inhibits CFTR mediated chloride secretion and thus, reduces the mucociliary clearance of pathogens.

## Results

### Cif promotes lysosome-mediated degradation of CFTR

**Figure 1:**
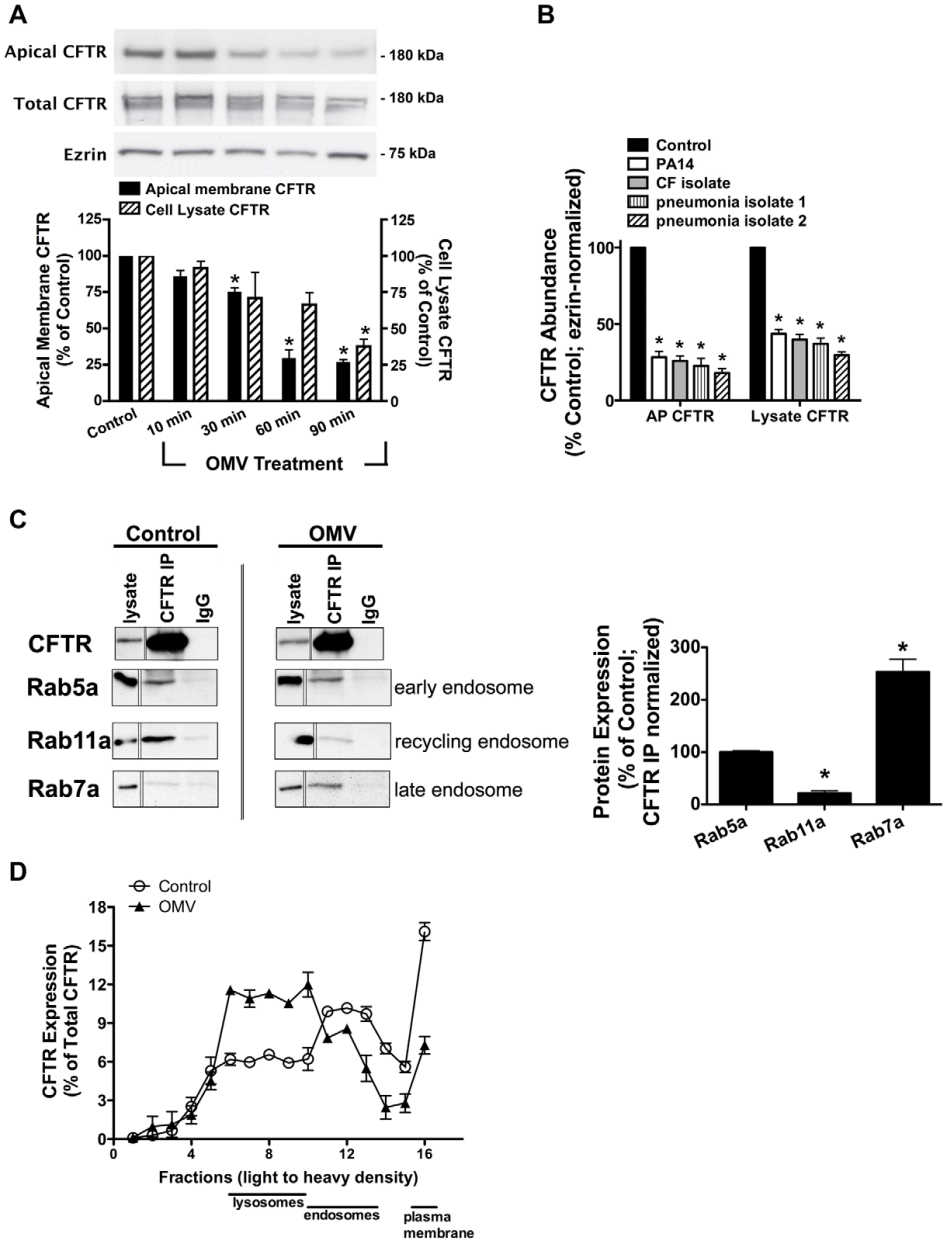
Cif redirects CFTR from recycling endosomes to the lysosomal, degradative pathway. A. Cif-containing OMV applied apically to polarized human airway epithelial cells resulted in a time-dependent reduction in wt-CFTR in cell lysates and the plasma membrane. Lysate CFTR was assessed by SDS-PAGE and western blot analysis. Cell surface biotinylation followed by SDS-PAGE and western blot analysis was performed to analyze the effect of Cif-containing OMV on apical membrane CFTR. Control was cells exposed to OMV lacking Cif, Δ*cif* OMV [2]. Solid black bars, apical membrane CFTR; striped bars, cell lysate CFTR. Quantitation of results found below representative blots. N = 3, * P<0.05 versus control. 95% confidence intervals (Apical membrane CFTR: control, 100 to 100; 10 min, 92.97 to 71.87; 30 min, 85.47 to 59.15; 60 min, 52.36 to 32.43; 90 min, 44.54 to 30.98; Cell lysate CFTR: control, 100 to 100; 10 min, 87.08 to 113.8; 30 min, 94.25 to 60.72; 60 min, 74.68 to 61.53; 90 min, 48.49 to 36.08). B. Purified OMV from *P. aeruginosa* (PA14), a recently isolated laboratory strain, and clinical isolates (CF and pseudomonal pneumonia) applied to airway epithelial cells for 90 minutes decreased cell lysate and apical membrane abundance of CFTR. Lysate CFTR was assessed by SDS-PAGE and western blot analysis. Cell surface biotinylation followed by SDS-PAGE and western blot analysis was performed to analyze the effect of clinical isolate OMV on apical membrane CFTR. N = 3, * P<0.05 versus control. 95% confidence intervals (Apical membrane CFTR: control, 100 to 100; PA14, 40.59 to 16.01; CF isolate, 36.47 to 15.22; pneumonia isolate 1, 43.51 to 1.545; pneumonia isolate 2, 30.49 to 5.482; Cell lysate CFTR: control, 100 to 100; PA14, 52.80 to 34.47; CF isolate, 50.77 to 28.91; pneumonia isolate 1, 52.89 to 21.02; pneumonia isolate 2, 38.62 to 20.69). C. Cif redirects CFTR from the recycling pathway to the lysosomal pathway. CFBE cells were incubated with OMV containing Cif, or with OMV lacking Cif (ΔCif-OMV), for 90 minutes and then endosomes were isolated as described in Methods. Subsequently, co-immunoprecipitation studies were conducted to determine the subcellular location of CFTR in control (ΔCif-OMV) and Cif OMV treated cells (left). Rab5a is a marker of early endosomes, Rab11 is a marker of recycling endosomes, and Rab7 is a marker of late endosomes. Thus co-immunoprecipitation of CFTR with: Rab5a identifies the amount of CFTR in early endosomes, Rab11a identifies the amount of CFTR in recycling endosomes, and Rab7 identifies the amount of CFTR in late endosomes. Quantification of data below for Rab immunoprecipitation with CFTR in the presence and absence of Cif OMV treatment, normalized for the amount of CFTR immunoprecipitated. N = 3, *P<0.05 versus control. 95% confidence intervals (Rab5a: 112.4 to 87.79; Rab7a: 41.44 to 1.746; Rab11a: 354.6 to 152.5). D. Optiprep gradient fractionation and differential centrifugation analysis of CFTR after 90 minutes incubation with Cif-containing OMV or control OMV (ΔCif-OMV) reveals a redistribution of CFTR from the plasma membrane and endosomes in control cells to lysosomes in Cif-treated cells. Data presented as CFTR detected in each fraction (by Western blot analysis) as a percentage of the total CFTR detected in all fractions. Plasma membrane, endosome and lysosomes designated in fractions based on Western blot of fraction for Na+/K+ ATPase, EEA-1 and Rab5a, and LAMP-1, respectively. Experiment performed 3 times.

To elucidate the mechanism for the reduction of apical membrane CFTR, we first examined the time course of the effect of Cif on the amount of CFTR in the plasma membrane. To this end, purified *P. aeruginosa* outer membrane vesicles (OMV) containing the Cif toxin were applied to the apical face of polarized human airway epithelial cells. *P. aeruginosa* OMV were isolated from an overnight culture and diluted to approximate the OMV produced by 10^8^ bacteria. A bacterial count of 10^8^ to 10^10^ is relevant because this is the bacterial density often detected in CF patient respiratory secretions [11]. After *P. aeruginosa* OMV treatment, CFTR was measured in cell lysates by Western blot analysis and in the apical plasma membrane by cell surface biotinylation followed by Western blot analysis. Cif rapidly (30–60 minutes) decreased the apical membrane abundance of CFTR and subsequently reduced CFTR protein levels in the cell lysate (Figure 1A). OMV purified from *P. aeruginosa* clinical isolates and applied to airway epithelial cells also significantly reduced the apical membrane and cell lysate abundance of CFTR (Figure 1B). By contrast, OMV isolated from *P. aeruginosa* lacking Cif had no effect on CFTR [2].

CFTR has a long half-life at the apical plasma membrane (8–24 hours) because it is efficiently recycled back to the plasma membrane after it is removed by endocytosis [12], [13], [14], [15]. Thus, the ability of Cif to rapidly (30–60 minutes) reduce CFTR abundance in human airway epithelial cells suggests that Cif enhances the endocytic removal of CFTR from the apical plasma membrane and/or reduces the recycling of endocytic vesicles containing CFTR back to the plasma membrane. In a previous publication, we demonstrated that Cif did not alter the endocytic rate of CFTR, but dramatically reduced the recycling of endocytic vesicles containing CFTR back to the plasma membrane [3]. In addition, our previous publication also demonstrated that Cif did not alter the abundance of other apical membrane proteins, like the transferrin receptor or the GPI-anchored protein, gp114 [3].

To begin to elucidate the mechanism whereby Cif altered CFTR trafficking, endosomes were isolated by density gradient purification from polarized human airway epithelial cells that had been treated with either OMV containing Cif or OMV from the *cif* mutant (control, in which the *cif* gene had been deleted). These experiments revealed that in control cells, CFTR co-immunoprecipitated with Rab5a and Rab11a, a finding consistent with previous reports that CFTR is localized primarily in early endosomal (Rab5a-labelled) and recycling endosomal (Rab11a-labelled) compartments (Figure 1C, [12], [13], [14], [16], [17], [18]). Although addition of Cif-containing OMV to polarized human airway epithelial cells did not change the amount of CFTR in early endosomes (Rab5a compartment), Cif dramatically shifted the distribution of CFTR from recycling endosomes (Rab11a-compartment) to the Rab7a, late endosomal compartment (Figure 1C). These results support the conclusion that Cif redirects CFTR from endosomes that recycle to the plasma membrane to a degradative pathway.

To investigate further the trafficking of CFTR in the presence of Cif, we followed the movement of CFTR through intracellular compartments via differential centrifugation and Optiprep gradient fractionation. In these experiments, apical membrane CFTR was biotinylated and the portion of CFTR that started at the apical membrane was tracked as a function of time after exposure to OMV containing or lacking Cif. In control cells (treated with OMV lacking Cif), most CFTR was present in the membrane fraction, but CFTR was also present in the endosomal and a small portion in the lysosomal fraction (Figure 1D). Addition of Cif-containing OMV reduced the amount of CFTR in the plasma membrane and in endosomes, and increased the amount of CFTR in the lysosomal compartment (Figure 1D). These results are consistent with the results presented in Figure 1C demonstrating that Cif reduces apical plasma membrane CFTR by redirecting CFTR from the recycling endocytic pathway (i.e., Rab 11a) to a lysosomal degradative pathway (i.e., Rab 7 and lysosomes).

### Cif targets CFTR to the lysosome via multi-ubiquitination

**Figure 2:**
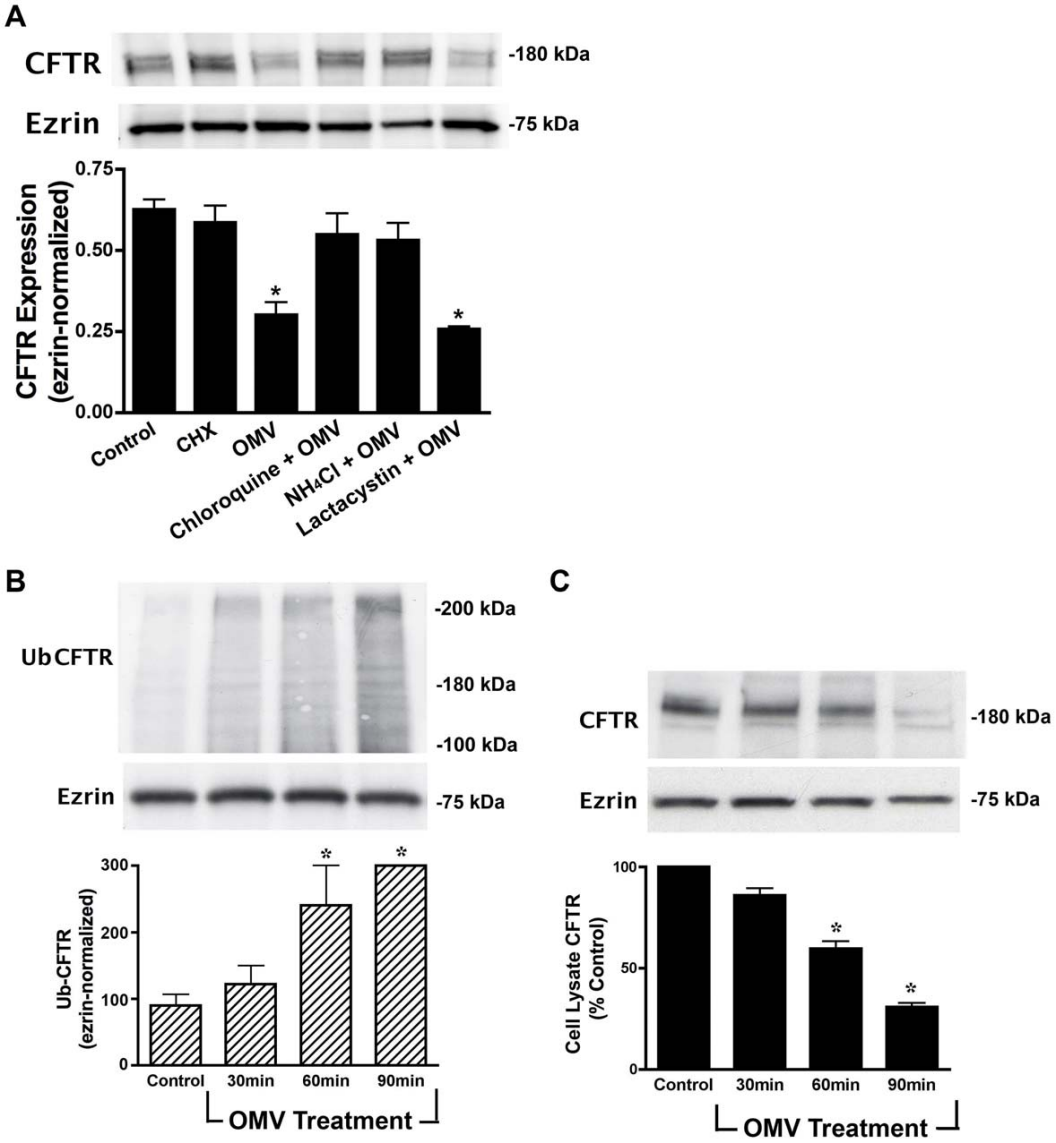
Cif induces the lysosomal-mediated degradation of CFTR in polarized human airway epithelial cells. A. Lysosomal inhibitors [chloroquine (200 µM) and NH_4_Cl (50 mM)] block the Cif-mediated degradation of CFTR, as assessed by Western blot analysis for CFTR in cells treated for 90 minutes with ΔCif-OMV (Control) or Cif-containing OMV, whereas the proteasomal inhibitor lactacystin (50 µM) had no effect on CFTR degradation. CHX, cycloheximide. All experiments, except control, were performed in the presence of 5 ng/ml cycloheximide to prevent protein synthesis, which could mask the effect of Cif OMV on CFTR degradation. Experiments were repeated at least 3 times, * p<0.05 versus control. 95% confidence intervals (Control, 0.55 to 0.71; CHX, 0.51 to 0.79; OMV, 0.21 to 0.34; CHQ+OMV, 0.49 to 0.69; NH4Cl+OMV, 0.48 to 0.65; Lactacystin+OMV, 0.22 to 0.30). B. Ubiquitinated CFTR was assessed by immunoprecipitation of CFTR and Western blotting for ubiquitin with the FK2 ubiquitin antibody in cells treated with ΔCif-OMV or Cif-containing OMV (90 min treatment). Western blotting with the FK1 ubiquitin antibody, that detects polyubiquitinated proteins, did not show a change in labeling after Cif treatment (data not shown). Ubiquitination experiments were performed in the presence of 200 µM chloroquine to allow detection of ubiquitinated CFTR, which is degraded too fast to be detected in the absence of a lysosomal inhibitor. Experiments were repeated at least 3 times, * p<0.05 versus control. 95% confidence intervals (Control, 92.47 to 107.45; 30 min, 98.48 to 138.56; 60 min, 175.25 to 280.34; 90 min, 289.10 to 302.83) C. Cif-containing OMV applied to airway epithelial cells elicited an increase in multi-ubiquitinated CFTR, concurrent with a reduction in cell lysate CFTR. Cells treated with ΔCif-OMV (control) or Cif-containing OMV (90 min treatment) were assessed via western blot analysis for CFTR abundance. Quantitation for Western blot experiments is presented with representative blots. All experiments were repeated at least 3 times, * p<0.05 versus control. 95% confidence intervals (Control, 98.45 to 103.76; 30 min, 90.47 to 76.29; 60 min, 72.19 to 53.73; 90 min, 43.10 to 31.39).

To provide additional support for the observation that Cif redirects CFTR to lysosomes for degradation, Cif-containing OMV were incubated in combination with control (vehicle) or the lysosomal inhibitors chloroquine or ammonium chloride. Both chloroquine and ammonium chloride reduced the Cif-mediated degradation of CFTR (Figure 2A). By contrast, the proteasomal inhibitor lactacystin had no effect on the Cif-mediated degradation of CFTR (Figure 2A). Thus, Cif increased the degradation of CFTR in the lysosome, a conclusion consistent with the results presented in Figures 1C and D.

The next series of experiments were conducted to elucidate the cellular mechanism whereby Cif redirected CFTR to lysosomes for degradation. First, we tested the hypothesis that Cif increased the amount of ubiquitinated CFTR, since it is known that ubiquitinated CFTR is degraded in the lysosome [10], [19]. Airway cells were treated with Cif-containing OMV for various time points, in the presence of a lysosomal inhibitor that prevents ubiquitinated CFTR from being degraded. CFTR was then immunoprecipitated and western blot analysis was performed using ubiquitin antibodies. Figure 2B demonstrates that Cif increased the amount of ubiquitinated CFTR with a time course that is concomitant with a decrease in the amount of CFTR (Figure 2C).

Several observations support the view that Cif increased the multi-ubiquitination of CFTR, rather than mono-ubiquitination or poly-ubiquitination. First, the ubiquitin antibody FK1, which only recognizes poly-ubiquitinated CFTR, did not identify ubiquitinated CFTR in the presence of Cif treatment (Figure S1). Second, the ubiquitin antibody, FK2, which recognizes mono- and multi-ubiquitinated proteins, recognized immunoprecipitated CFTR (Figure 2B). Third, the molecular weight of ubiquitinated CFTR in the presence of Cif increased by ∼40 kDa, an amount greater than 8 kDa, the molecular weight of a single ubiquitin moiety. Thus, taken together these results are consistent with the conclusion that Cif enhances the degradation of CFTR primarily by increasing the amount of multi-ubiquitinated CFTR, and its subsequent degradation in the lysosome.

### Cif inactivates USP10 in the early endosome

Cif may increase the amount of multi-ubiquitinated CFTR, and thereby its degradation in lysosomes, by activating an E3 ligase and/or by inactivating a DUB. To determine the mechanism by which Cif increases the amount of multi-ubiquitinated CFTR, Cif was applied to polarized human airway epithelial cells in OMV, followed by immunoprecipitation of Cif to identify Cif-interacting proteins. Mass spectrometry of the immunoprecipitated proteins revealed interaction of Cif with several DUBs, including Ubiquitin Specific Protease-10 (USP10) and USP34 (data not shown). We previously reported that the DUB USP10 deubiquitinates CFTR in early endosomes of human airway epithelial cells [10].

**Figure 3:**
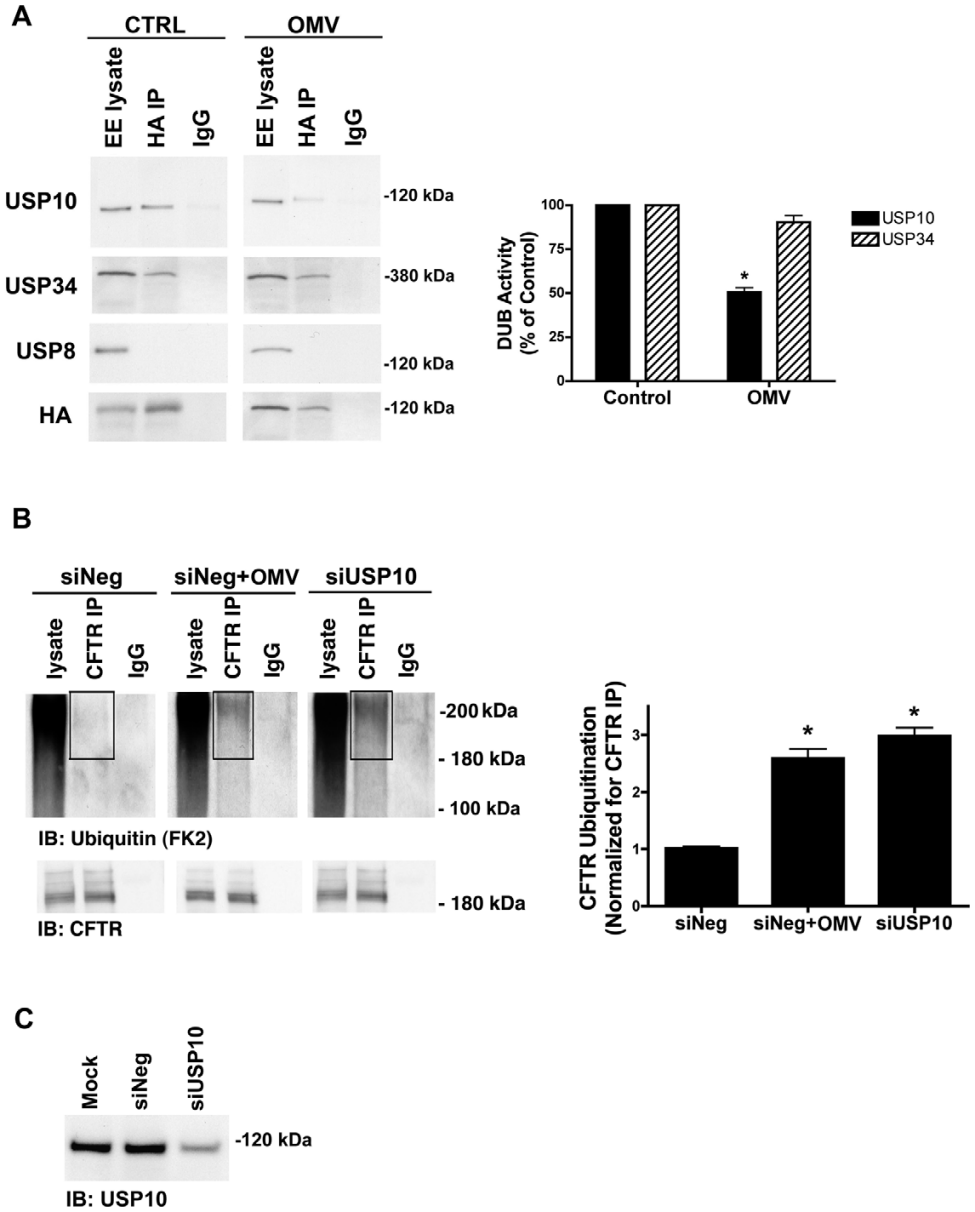
Cif selectively inhibits USP10 activity in early endosomes. A. Cif-containing OMV reduced the activity of a 110 kDa deubiquitinating enzyme (DUB) as assessed by a DUB activity assay in cells treated with ΔCif-OMV (Control) or Cif-containing OMV (15 min treatment; see Methods and [20], [21], [45]). The DUB activity assay employs a HA-UbVME probe that forms an irreversible, covalent bond only with active DUBs. Identification of DUBs covalently linked to the HA-UbVME probe was achieved by immunoprecipitation of the HA-UbVME-DUB complex using an anti-HA monoclonal antibody followed by SDS-PAGE and western blot analysis. The 110 kDa DUB was identified as USP10 by Western blot. USP34 and USP8 were also identified in early endosomes by western blot, however, the DUB activity assay revealed that USP34 was active, but its activity was not altered by Cif. By contrast, USP8 activity was not detected. Quantitation for all western blot experiments is presented to the right. All experiments were repeated at least 3 times, * p<0.05. 95% confidence intervals (USP10: control, 100 to 100; OMV, 61.66 to 39.20; USP34: control 100 to 100; OMV, 136.70 to 44.31). B. siRNA knockdown of USP10 abundance recapitulates the Cif-mediated increase in CFTR ubiquitination, as assessed by immunoprecipitation of CFTR and western blotting for multi-ubiquitinated CFTR in Cif OMV or siRNA-USP10 treated cells. Experiments were performed in the presence of 200 µM chloroquine to allow accumulation and detection of ubiquitinated CFTR. siNeg, nonspecific, scrambled siRNA served as negative control for siRNA transfections. IgG, immunoprecipitation using a non-immune IgG was used as a negative control. Black boxes highlight multi-ubiquitinated CFTR on blots. Quantitation for western blot experiments is presented beside representative blots. All experiments were repeated at least 3 times, * p<0.05. 95% confidence intervals (siNeg, 0.88 to 1.14; siNeg+OMV, 1.87 to 3.30; siUSP10, 2.35 to 3.61). C. siRNA knockdown of USP10 reduces USP10 protein levels in airway cells, as assessed by western blot analysis. A USP10 representative western blot is shown for cell lysates from experiments in Figure 3B, detecting ubiquitinated CFTR.

To determine if Cif inhibits the activity of USP10, and thereby increases the amount of ubiquitinated CFTR, we used a DUB activity assay to measure USP10 activity in early endosomes (EE) of airway epithelial cells [10], [20], [21], [22]. The DUB activity assay employs a HA-UbVME probe that forms an irreversible, covalent bond only with active DUBs. Identification of DUBs covalently linked to the HA-UbVME probe was achieved by immunoprecipitation of the HA-UbVME-DUB complex using an anti-HA monoclonal antibody followed by SDS-PAGE and western blot analysis for the DUB of interest. Using this assay, we demonstrated that USP10 activity was inhibited 49±4% by Cif (Figure 3A). Cif did not alter the activity of other EE-resident DUBs including USP8 or USP34, thereby demonstrating the specificity of Cif in EE for USP10 (Figure 3A). Moreover, silver stain analysis of the DUB activity assay revealed that Cif does not alter the activity of any DUBs in addition to USP10 (data not shown).

To provide additional support for the hypothesis that Cif inactivation of USP10 is responsible for the increase in the amount of ubiquitinated CFTR and its lysosomal degradation, we examined the amount of multi-ubiquitinated CFTR following siRNA knockdown of USP10. siRNA-mediated reduction of USP10 protein expression (by 76±4%, Figure 3C) increased the amount of ubiquitinated CFTR (Figure 3B, [10]). These data are consistent with the view that Cif reduces USP10 activity and thereby increases the amount of multi-ubiquitinated CFTR and its degradation in the lysosome.

### Cif stabilizes USP10 and G3BP1 interaction to inhibit USP10 activity

**Figure 4:**
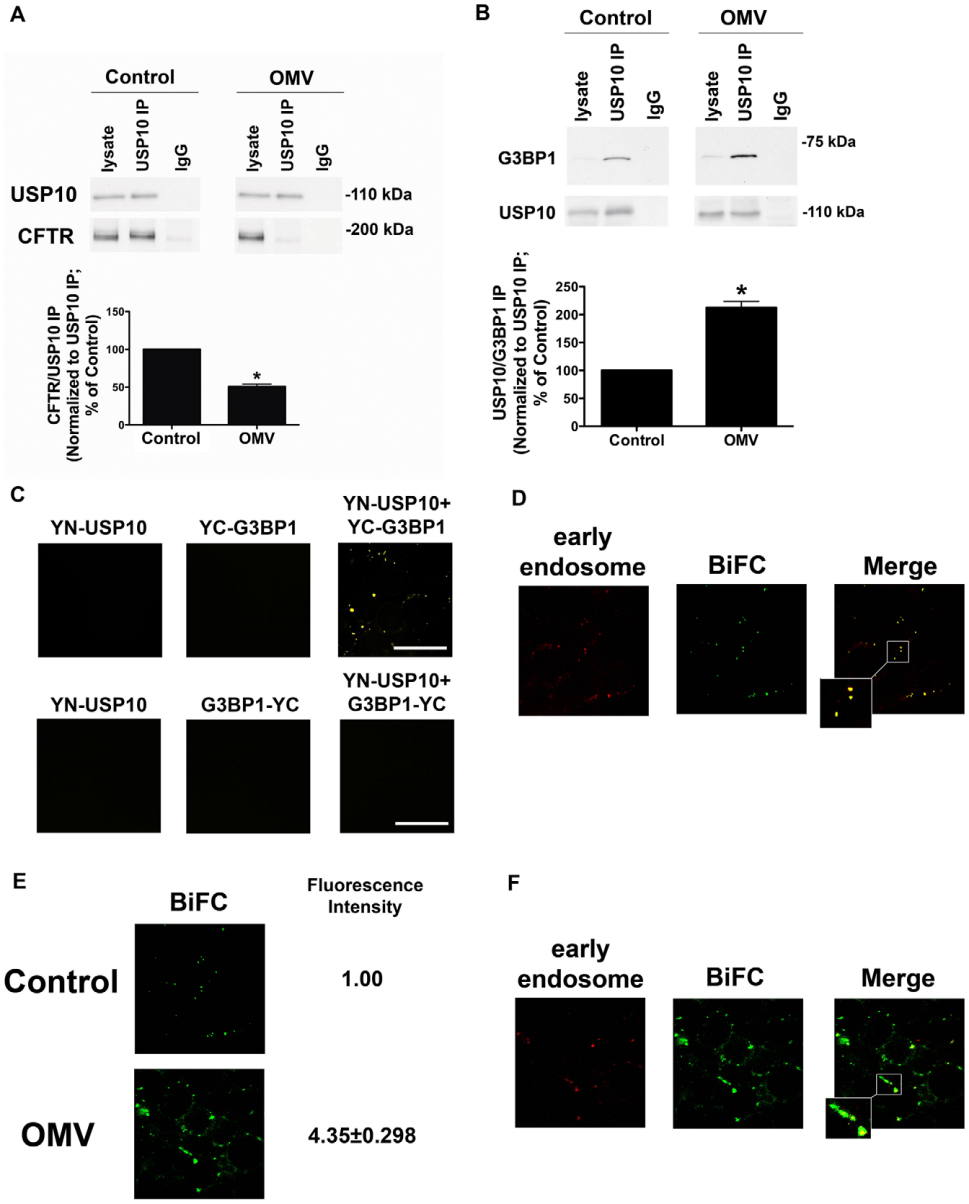
Cif disrupts CFTR-USP10 interaction by stabilizing the USP10-G3BP1 interaction. A. Cif-containing OMV decrease the interaction of CFTR with USP10 in the early endosomes of airway epithelial cells, as assessed by immunoprecipitation of USP10 from EE and western blot analysis for CFTR in cells treated with ΔCif-OMV or Cif-containing OMV (90 min treatment). Quantitation of USP10-CFTR immunoprecipitation, normalizing for USP10 immunoprecipitation efficiency, is presented at the bottom of the panel. IgG, immunoprecipitation using a non-immune IgG was used as a negative control. All experiments were repeated at least 3 times, * P<0.05 versus control. 95% confidence intervals (Control, 100 to 100; OMV 56.41 to 48.29). B. Cif-containing OMV increase the interaction of G3BP1 with USP10 in the early endosomes of airway epithelial cells, as assessed by immunoprecipitation of USP10 from an early endosomal fraction and western blot analysis for G3BP1. Control cells were treated with ΔCif-OMV. Quantitation of USP10-G3BP1 immunoprecipitation, normalizing for USP10 immunoprecipitation efficiency, is presented at the bottom of the panel. IgG, immunoprecipitation using a non-immune IgG was used as a negative control. All experiments were repeated at least 3 times, * p<0.05 versus control. 95% confidence intervals (Control, 100 to 100; OMV 164.7 to 260.5). C. Bimolecular fluorescence complementation experiments confirm G3BP1 and USP10 interaction in airway epithelial cells. Airway epithelial cells were transfected with expression constructs 1-154YFP-USP10 (YN-USP10) and 155-238YFP-G3BP1 (YC-G3BP1) individually and in combination, as well as YN-USP10 and G3BP1-YC individually and in combination. Six other combinations of USP10 and G3BP1 constructs with different orientation of the half YFP protein in the fusion proteins (N- or C-terminus) achieved varying degrees of BiFC fluorescence (data not shown). Experiments in Figures 4d–f were performed with the constructs yielding maximal BiFC fluorescence intensity under control conditions, YN-USP10 and YC-G3BP1 (Figure 4c). D. Bimolecular fluorescence complementation experiments confirm G3BP1 and USP10 interaction in the early endosomal compartment of airway epithelial cells. Airway epithelial cells were transfected with expression constructs YN-USP10 and YC-G3BP1 and subsequently infected with a baculovirus Rab5a-eRFP construct, which is a marker of early endosomes (red). Infection with the baculovirus expressing the empty vector had no effect on cellular morphology, and fluorescence in the red channel in cells infected with this baculovirus was similar to background (data not shown). E. Bimolecular fluorescence complementation experiments demonstrate an increased G3BP1-USP10 interaction in airway epithelial cells treated with Cif-containing OMV. CFBE cells were transfected with expression constructs YN-USP10 and YC-G3BP1 and treated with Cif-containing or ΔCif-OMV (Control). Fluorescence intensity is expressed as fold change from control. F. Bimolecular fluorescence complementation experiments reveal the increased G3BP1 and USP10 interaction with Cif treatment is localized primarily to the early endosomal compartment of airway epithelial cells. Airway epithelial cells were transfected with expression constructs YN-USP10 and YC-G3BP1 and subsequently infected with a baculovirus Rab5a-eRFP construct, which is expressed in early endosomes (red). Maximum intensity projections are presented for all confocal microscopy images. Scale bars are equal to 20 µm. Ten fields were imaged per experiment and experiments were repeated at least three times.

The next set of experiments was designed to elucidate how Cif inhibits USP10 activity. Three observations suggest that Cif may inhibit USP10 activity by stabilizing the interaction between USP10 and G3BP1, which inhibits USP10 DUB activity, and also reduces the interaction between USP10 and CFTR. First, published studies have shown that USP10 and G3BP1 interact in yeast and mammalian systems [23], [24], [25]. Second, in U2OS bladder cancer cells, the interaction between G3BP1 and USP10 inactivates the deubiquitinating enzyme activity of USP10 [23]. Third, our preliminary mass spectrometry experiments revealed that Cif and G3BP1 interact (data not shown). Thus, studies were conducted to determine if Cif inhibits USP10 activity by stabilizing the interaction between USP10 and G3BP1, and by reducing the interaction between USP10 and CFTR. As shown in Figure 4A, USP10 interacts with CFTR in the early endosomes isolated from airway epithelial cells, and Cif reduces this interaction by 50±6%. Moreover, Cif increased the interaction between G3BP1 and USP10 by 210±12% (Figure 4B). Thus, these observations reveal that Cif stabilizes an interaction between USP10 and G3BP1, and also reduces the interaction between USP10 and CFTR.

To provide additional support for the immunoprecipitation studies demonstrating that Cif increases the interaction between G3BP1 and USP10 in the early endosomal compartment, we performed bimolecular fluorescence complementation (BiFC) studies. BiFC utilizes two half yellow fluorescent protein (YFP) sequences fused to two hypothetical interacting proteins (USP10 and G3BP1). If two fusion proteins interact (e.g., 1-154YFP-USP10 (YN-USP10) and 155-238YFP-G3BP1 (YC-G3BP1)), a full YFP protein is formed and fluorescence is detected via confocal microscopy [26]. Moreover, by co-transfecting cells with organelle markers, it is possible to identify the compartment where USP10 and G3BP1 interact. In control experiments transient transfection of any one of the eight half-YFP constructs alone in airway epithelial cells did not result in fluorescence in the yellow channel, as expected (Figure 4C and not shown). By contrast, co-transfection of both constructs (YN-USP10 and YC-G3BP1) yielded YFP fluorescence (Figure 4C). Combinations of USP10 and G3BP1 constructs with different orientation of the half YFP protein in the fusion proteins (N- or C-terminus) achieved varying degrees of BiFC fluorescence (Figure 4C and not shown). Experiments in Figures 4d–f were performed with the constructs yielding maximal BiFC fluorescence intensity under control conditions, YN-USP10 and YC-G3BP1 (Figure 4C).

To provide additional support for our immunoprecipitation studies that the interaction between USP10 and G3BP1 occurs in early endosomes (Figure 4B), BiFC experiments were performed in cells transduced with a baculovirus system expressing eRFP-labeled Rab5a (an early endosomal protein) to label early endosomes. Co-localization of the BiFC signal with the early endosomal marker was quantified by intensity correlation analysis using Nikon Elements Software. Mander's overlap coefficients of 0.84±0.06 demonstrated a high degree of co-localization of the USP10-G3BP1 pair with early endosomes (Figure 4D). Treatment of co-transfected (YN-USP10 and YC-G3BP1) airway epithelial cells with Cif-containing OMV resulted in an increase in BiFC signal, confirming an increased (4.35±0.30 fold) interaction between USP10 and G3BP1 in the presence of Cif (Figure 4E) compared to cells treated with control (Cif mutant OMV). Co-localization studies revealed that the BiFC signal (USP10-G3BP1 interaction) was localized to the early endosomal compartment (Rab5a) in airway epithelial cells treated with Cif-containing OMV (Figure 4F, Mander's overlap coefficient of 0.76±0.11). These studies support the conclusion that Cif stabilizes the interaction between USP10 and G3BP1 in the early endosomes.

**Figure 5:**
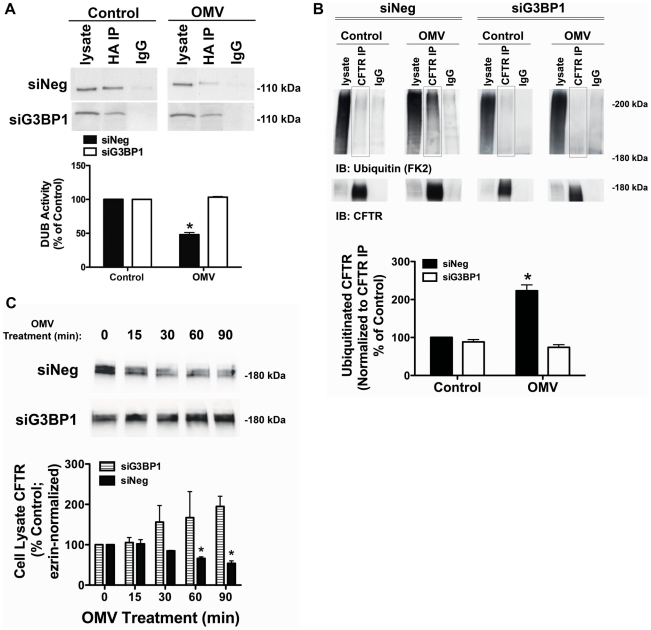
siRNA knockdown of G3BP1 eliminates Cif inhibition of USP10. A. siRNA knockdown of G3BP1 prevented the Cif-mediated inhibition of USP10 deubiquitinating enzyme activity in the EE fraction, as assessed with the DUB activity assay (using the HA-UbVME probe). Control cells were treated with Δ*cif*-OMV. Quantification of USP10 DUB activity is presented below. Experiments were repeated at least 3 times, * P<0.05 versus control. 95% confidence intervals (siNeg: Control, 100 to 100; OMV, 61.12 to 34.87; siG3BP1: Control, 100 to 100; OMV, 107.8 to 98.69). B. siRNA G3BP1 prevented the Cif-mediated increase in CFTR multi-ubiquitination, as assessed by immunoprecipitation of CFTR followed by western blotting for ubiquitin (clone FK2 antibody). Experiments were performed in the presence of 200 µM chloroquine to block the degradation of CFTR to allow detection of ubiquitinated CFTR. Black boxes highlight multi-ubiquitinated CFTR on blots. IgG, immunoprecipitation using a non-immune IgG was used as a negative control. Quantification for all western blot experiments is presented at the bottom of the panel. Experiments were repeated at least 3 times, * P<0.05 versus control. 95% confidence intervals (siNeg: Control, 102.6 to 98.30; OMV, 173.9 to 272.4; siG3BP1, Control, 108.2 to 68.72; OMV 96.27 to 51.79). C. siRNA G3BP1 prevented the Cif-mediated lysosomal degradation of CFTR, as assessed by western blot analysis. A similar result was observed for apical membrane expression of CFTR (data not shown). Quantification for all western blot experiments is presented below representative blots. Experiments were repeated at least 3 times, * P<0.05 versus control. 95% confidence intervals (siNeg: Control, 100 to 100; 15 min, 147.1 to 57.14; 30 min, 89.36 to 80.44; 60 min, 73.27 to 39.17; 90 min, 60.72 to 27.02; siG3BP1: Control, 100 to 100; 15 min, 159.4 to 51.42; 30 min, 60.22 to 232.5; 60 min, 110.8 to 334.9; 90 min, 86.24 to 303.4).

Finally, if Cif stabilizes an inhibitory interaction between G3BP1 and USP10, silencing G3BP1 should reduce the Cif-induced decrease in USP10 activity as well as the Cif-induced increase in ubiquitinated CFTR, and its degradation in lysosomes. siRNA-mediated knockdown of G3BP1 protein expression (by 50%±3%) eliminated the ability of Cif to inhibit USP10 activity (Figure 5A). If G3BP1 knockdown prevented the Cif-mediated inhibition of USP10 activity, we would predict that knockdown of G3BP1 would also eliminate the increase in multi-ubiquitinated CFTR and degradation of CFTR induced by Cif. Knockdown of G3BP1 did, in fact, block the Cif-mediated increase in the amount of multi-ubiquitinated CFTR (Figure 5B) and enhanced lysosomal degradation of CFTR (Figure 5C). Notably, siRNA-mediated knockdown of G3BP1 significantly increased the abundance of CFTR, most likely because endogenous G3BP1 inhibits USP10 (Figure 5C). Two additional siRNA target sequences for G3BP1 also abrogated the Cif-mediated degradation of CFTR (Figure S2). Taken together, these data confirm our hypothesis that Cif, by enhancing G3BP1 interaction with USP10, inhibits the ability of USP10 to interact with, and deubiquitinate CFTR.

## Discussion

**Figure 6:**
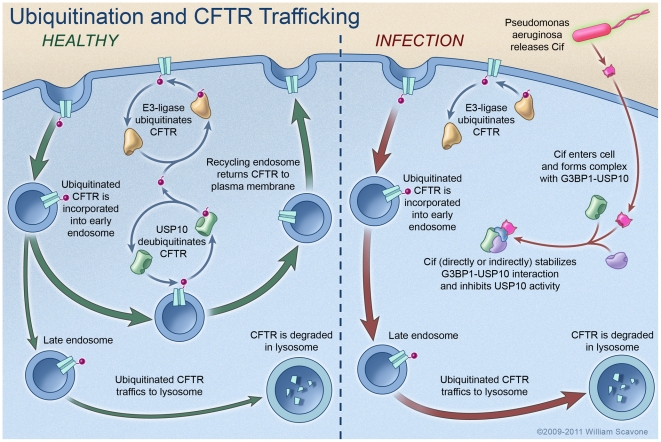
Model proposing a mechanism whereby Cif inhibits USP10 activity and reduces the apical membrane abundance of CFTR in polarized, human airway epithelial cells. In the healthy state (i.e., not infected with *P. aeruginosa*), CFTR constitutively cycles in and out of the apical plasma membrane via trafficking through the early endosomal and recycling endosomal compartments. The secretion of Cif in OMV by *P. aeruginosa* redirects CFTR from the recycling pathway to the lysosomal, degradative pathway via an increase in the amount of multi-ubiquitinated CFTR. Cif inhibits the DUB activity of USP10 to increase the amount of ubiquitinated CFTR by stabilizing an inhibitory interaction between USP10 and G3BP1, and by decreasing the interaction between CFTR and USP10. It is still not known whether Cif directly or indirectly interacts with USP10 and G3BP1. These effects of Cif reduce the deubiquitination of CFTR in the early endosome and increase the lysosomal degradation of CFTR. Reduced apical membrane abundance of CFTR reduces Cl^-^ secretion [3], [4], and is predicted to diminished mucociliary clearance, severely compromising the immune defenses of the lung [42], [43].

To our knowledge this is the first report demonstrating that a bacterial toxin, Cif (PA2934), regulates a host protein (USP10) involved in ubiquitination and lysosomal degradation of an ion channel (CFTR), and thereby modulates the ability of airway epithelial cells to secrete chloride, an important component of the innate immune response in the lung. The data in this report demonstrates that Cif alters the intracellular trafficking of CFTR, redirecting CFTR from endosomes where CFTR recycles to the plasma membrane to lysosomes where CFTR is degraded. Our data support the conclusion that Cif stabilizes an inhibitory effect of G3BP1 on USP10, thereby reducing its ability to deubiquitinate CFTR and increasing the degradation of CFTR in lysosomes (Figure 6). These data suggest that the ability of *P. aeruginosa* to chronically infect the lungs of patients with COPD, pneumonia, CF, and bronchiectasis is due in part to the secretion of OMV containing Cif, which inhibits CFTR-mediated chloride secretion and thus, diminishes the clearance of respiratory pathogens by the mucociliary escalator.

Ubiquitin modification of proteins regulates numerous cell processes, including protein degradation, intracellular protein trafficking, and cell signaling [27]. Ubiquitination and deubiquitination are dynamic and regulated processes; over 1000 E3 ligases, which attach ubiquitin moieties to substrate proteins, are encoded by the human genome, whereas ∼90 DUBs remove ubiquitin from substrate proteins [6], [7]. Thus, the amount of an ubiquitinated protein, and thereby the amount of the protein that is degraded in lysosomes or the proteasome, is regulated by the balance between the activity of E3 ligases and DUBs. Importantly, the abundance of a given protein is thought to be regulated by a small subset of E3 ligases and DUBs [6], [7].

At the present time, two E3 ligases (Nedd4-2 and c-Cbl) have been shown to regulate CFTR trafficking. In pancreatic cells, Nedd4-2 regulates CFTR abundance [28], but preliminary studies from our laboratory demonstrate that silencing Nedd4-2 does not alter the amount of CFTR in human airway epithelial cells (unpublished data). Recently, we demonstrated that c-Cbl ubiquitinates CFTR in airway epithelial cells [29]; however, siRNA studies from our laboratory demonstrated that siRNA knockdown of c-Cbl did not inhibit the Cif-mediated increase in CFTR degradation (unpublished data). Thus, it is unlikely that Cif increases the amount of ubiquitinated CFTR by activating Nedd4-2 or c-Cbl. However, it cannot be ruled out that Cif may be activating an unknown E3 ligase to increase the amount of ubiquitinated CFTR. Additional experiments, beyond the scope of this study, are required to determine if other E3 ligases ubiquitinate CFTR in airway epithelial cells and to determine if Cif regulates the activity of these E3 ligases.

Approximately 90 DUBs remove ubiquitin from target proteins [6], [7]. The effect of these ligases and DUBs are known to be highly specific. For example, in this and a previous study we observed that neither USP34, nor USP8 deubiquitinate CFTR, and only USP10 activity was inhibited by Cif [10], [30]. Our results suggest a model whereby in steady-state conditions, USP10 activity is regulated by dynamic interactions between its target protein, CFTR and its negative regulator, G3BP1. Upon *P. aeruginosa* infection and OMV delivery of Cif into host cells, the interaction of USP10 with its negative regulator, G3BP1, is stabilized and USP10 is sequestered from interaction with CFTR. Thus, CFTR remains multi-ubiquitinated in early endosomes and is targeted for lysosomal degradation (Figure 6). It is not currently known which ubiquitin linkages USP10 targets, but in our study it appears to target the multi-ubiquitinated CFTR. A previous study reported that G3BP1 interacts directly with the N-terminus of USP10, a common protein-protein interaction domain in the USP family of DUBs for regulation of DUB activity [23]. While our mass spectrometry data revealed an interaction between Cif and G3BP1, additional studies are needed to determine if the interaction is direct or indirect, and to elucidate how Cif stabilizes the inhibitory protein complex between USP10 and G3BP1.

Given the importance of the host ubiquitin degradation system in regulating basic cell biology, it is not surprising that many pathogens have evolved to target the ubiquitin pathway to promote their colonization of the host. Pathogen effects on E3 ligases to modify host cell function are well documented, but only recently has pathogen manipulation of the deubiquitinating machinery of the host been investigated [6], [7], [8], [9], [31]. Several bacterial species have been shown to encode deubiquitinating enzymes, the majority playing a role in dampening the host inflammatory response [6], [32], [33], [34], [35]. To date, one other host DUB (i.e., in addition to USP10) has been targeted by a bacterial species. The host-encoded DUB, Cylindromatosis (CYLD), is regulated indirectly by bacterial pathogens through changes in its gene expression, but a mechanism for the altered gene expression has yet to be reported [36], [37], [38], [39]. Infection with *Haemophilus influenzae* or *Eschericia coli* induces CYLD expression, which down-regulates the NF-κB inflammatory pathway. CYLD -/- mice have a hypersensitivity to infection with both *Haemophilus influenzae* and *Eschericia coli* [37], [38], [39]. On the other hand, the CYLD -/- mice experience acute lung injury and increased lethality in response to *Streptococcus pneumoniae* infection [36]. These opposing effects of CYLD DUB activity in response to different pathogens suggest a potential complexity in targeting host DUBs for therapeutic purposes to combat infection.

To enable therapeutic development targeting bacterial effector proteins, and thereby bacterial infections, a better understanding of the mechanism of action of the bacterial effectors is required. The crystal structure of the Cif toxin has recently been solved and shows homology with the α/β hydrolase family of bacterial enzymes [40], [41]. Cif catalyzes the hydrolysis of epoxide compounds, with specific activity against epibromohydrin and cis-stilbene oxide. Interestingly, mutations to the active site of Cif that reduce epoxide hydrolase activity also reduce the effect of Cif on CFTR degradation [40]. Current studies are underway to elucidate the mechanism by which the epoxide hydrolase activity of Cif promotes the inactivation of USP10, via enhancement of G3BP1 interaction, leading to the degradation of CFTR.

The data in this manuscript is relevant to clinical infections by *P. aeruginosa* since the Cif toxin is expressed by clinical isolates of *P. aeruginosa* and in OMV isolated from CF and pseudomonal pneumonia patients ([4], unpublished data). Accordingly, taken together with previous studies on Cif, the data in this paper are consistent with the view that Cif-mediated reductions in CFTR abundance (∼60%) and chloride secretion (∼60%) by human airway epithelial cells [4] would be expected to reduce mucociliary clearance in the airway, a critical mechanism of the innate immune response to eliminate *P. aeruginosa* and other pathogens from the airway of patients with COPD, ventilator-associated pneumonia, CF and bronchiectasis [42], [43]. In addition to the clinical relevance of the Cif toxin on host mucociliary clearance and innate immune defense this study reports data identifying Cif as the first bacterial toxin that inactivates a host DUB. Understanding the mechanism by which bacterial toxins alter host cell biology provides the basis for therapeutic development to inhibit toxin function and potentially reduce bacterial pathogenesis.

## Methods

### Cell culture

The role of Cif in CFTR degradation was studied in human airway epithelial cells (CFBE41o- cells, homozygous for the ΔF508 mutation) stably expressing wt-CFTR (hereafter called airway epithelial cells). The derivation and characterization of these cells have been described in detail by several laboratories [13], [44]. Airway epithelial cells between passages 18 and 27 were grown and polarized in an air-liquid interface culture at 37°C for 6–9 days, as described [13].

### Identification of active DUBs

To identify active DUBs in airway epithelial cells we used a chemical probe screening approach designed and described in detail by Dr. Hidde Ploegh [20], [21], [45] and recently published by our laboratory [10]. The specificity of the HA-UbVME probe for active DUBs was confirmed with the addition of N-ethylmaleimide (10 µM), which inhibits cysteine protease DUBs, during the labeling reaction [20], [21], [45].

### Isolation of early endosomes

To determine if USP10 is expressed in early endosomes, differential centrifugation and fractionation techniques were used to isolate early endosomes from CFBE cells using a protocol adapted from Butterworth *et al.* [46]. Briefly, polarized CFBE cells, grown on 24 mm permeable membrane supports, were scraped into phosphate-buffered saline, pelleted, and resuspended in 600 µl of HEPES buffer (250 mM sucrose, 10 mM HEPES, 0.5 mM EDTA at pH 7.4 containing protease inhibitors (Roche Diagnostic Corp., Indianapolis, IN)). The cells were homogenized with a Dounce homogenizer and passed through a 22-gauge needle 20 times. Following a low speed spin (3,000× g), the post-nuclear supernatant was diluted 1∶1 with 62% sucrose in HEPES buffer and placed at the bottom of a 4.4 ml ultracentrifuge tube (Sorvall, Ashville, NC). 1.5 ml of 35% sucrose in HEPES buffer was layered on top followed by 1.5 ml of 25% sucrose in HEPES buffer and 0.5 ml of HEPES buffer. The gradients were centrifuged in a TH-660 rotor at 167,000× g for 75 min at 4°C, and the interfaces were collected to isolate the early endosomal fractions. Western blot analysis for various Rab GTPases was used to confirm purity of the early endosomal fraction [10].

### Optiprep gradient fractionation of plasma membrane, endosome, and lysosome compartments

To assess the trafficking of Cif through the endocytic trafficking pathway, we used differential centrifugation and an Optiprep continuous gradient to separate the plasma membrane, endosome, and lysosome fractions from airway epithelial cells, a protocol adapted from a previous study [47]. Plasma membrane (Na/K ATPase), endosome (early endosomal antigen-1, EEA-1), and lysosome (LAMP-1) resident proteins were used to identify these compartments in the fractionations to identify the localization of CFTR.

### Ubiquitination assay

To assess the amount of ubiquitinated CFTR in airway epithelial cells, a protocol was adapted from Urbe *et al*. [48] and recently published by our laboratory [10].

### Immunoprecipitation

To determine if CFTR interacts with USP10 in the early endosome (EE), USP10 was immunoprecipitated from EE fractions isolated from airway epithelial cell lysates by methods described previously in detail [12].

### Biochemical determination of cell surface CFTR

The biochemical determination of plasma membrane CFTR was performed by domain selective cell surface biotinylation using EZ-Link Sulfo-NHS-LC-Biotin (Pierce), as described previously in detail [49].

### RNA-mediated interference

USP10 and G3BP1 protein expression was selectively reduced using siRNA purchased from Qiagen (Valencia, CA), by methods described previously [12]. In brief, airway epithelial cells were seeded at 0.1×10^6^ on 24 mm Transwell permeable membrane supports and on day 4, post-seeding, cells were transfected with HiPerfect transfection reagent according to the manufacturer's protocol (Qiagen, Valencia, CA). Sequences for siRNAs are: siUSP10 sense 5′ CACAGCUUCUGUUGACUCUTT 3′; siG3BP1 #1 sense 5′ GGAGGAGUCUGAAGAGATT 3′; siG3BP1 #2 sense 5′ CCCUGGUUCCAACAGAAUGTT 3′; siG3BP1 #3 sense 5′ GAAAGAAAUCCACAGGAAATT 3′; siNegative scrambled sense 5′ UUCUCCGAACGUGUCACGU 3′. Cells were studied on day 8 post-seeding (i.e., 4 days after transfection with siRNA).

### Production of fluorescent fusion proteins

The sequences encoding YFP (1–154) (yellow fluorescent protein-1–154-peptide) and YFP (155–238) were kindly provided by Dr. Tom K. Kerppola (University of Michigan Medical School, Ann Arbor, MI, U.S.A.) [50], [51] and cloned onto the N- or C-terminal end of the human USP10 to produce *USP10*-*YN*, *USP10*-*YC*, *YN*-*USP10 and YC-USP10*. The human G3BP1 was also fused to the same YFP sequences to produce *G3BP1*-*YN*, *G3BP1*-*YC*, *YN*-*G3BP1 and YC-G3BP1*. The constructs were purchased from OriGene (pCMV6 vectors, Rockville, MD) and verified by DNA sequencing.

### BiFC confocal microscopy

CFBE-WT cells seeded at 0.1×10^6^ on collagen-coated, glass-bottom MatTek dishes, were transfected with 1 µg of a single USP10 and G3BP1 BiFC construct using the Effectene transfection reagent, according to manufacturer's protocol (Qiagen, Valencia, CA). All combinations of the USP10 and G3BP1 BiFC fusion proteins were transfected in pilot experiments and the single combination demonstrating maximum BiFC fluorescence was used in remaining experiments (YN-USP10 and YC-G3BP1). Two days post-transfection, cells were infected with a baculovirus expressing a RFP-Rab5a plasmid (Organelle Lights Endosomes-RFP, Molecular Probes, Invitrogen), according to the manufacturer's instructions. Samples were incubated in the presence or absence of Cif-containing OMV for 15 min at 37°C and then fixed with 4% paraformaldehyde in PBS for imaging. Z-stack images (0.4 µm sections) of labeled cells were acquired with a Nikon Sweptfield confocal microscope (Apo TIRF 100x oil immersion 1.49 NA objective) fitted with a QuantEM:512sc camera (Photometrics, Tuscon, AZ) and Elements 2.2 software (Nikon, Inc.). YFP fluorescence emission was measured at 535/30 nm and the fluorescence of Rab5a-RFP, a red fluorescent protein, was measured at 610/30 nm. Experiments were repeated three times, with ten fields imaged for each experiment.

### Antibodies and reagents

The antibodies used were: mouse anti-ezrin antibody, mouse anti-G3BP1, mouse anti-GFP antibody (BD Biosciences, San Jose, CA); mouse anti-HA antibody (Santa Cruz Biotechnology, Santa Cruz, CA); mouse anti-Ubiquitin antibodies (clones FK2 and FK1) (BioMol, Plymouth Meeting, PA); rabbit anti-USP10 antibody, rabbit anti-USP34, rabbit anti-USP8 (Bethyl Laboratories, Montgomery, TX); horseradish peroxidase-conjugated goat anti-mouse and goat anti-rabbit secondary antibodies (Bio-Rad, Hercules, CA). CFTR antibodies were used as described previously [10]. All antibodies and reagents were used at the concentrations recommended by the manufacturers or as indicated in the figure legends.

### Statistical analysis

Statistical analysis of the data was performed using Graphpad Prism version 4.0a for Macintosh (Graphpad, San Diego, CA). When appropriate, experimental triplicates were performed and all replicates were expressed as a percentage of control before the mean was determined. Means were compared using a t-test or ANOVA followed by Tukeys test, as appropriate. P<0.05 was considered significant. Data are expressed as the mean ± SEM. To show data distribution, 95% confidence intervals are presented in the figure legends.

### Accession numbers

Cif (PA2934, NP 251624.1); USP10 (NP 005144.2); G3BP1 (NP 005745.1); Rab5a (NP 004153.2); Rab7a (NP 004628.4); Rab11a (NP 004654.1); CFTR (NP 000483.3)

## Supporting Information

Figure S1 Cif does not alter the poly-ubiquitination of CFTR in polarized human airway epithelial cells. Poly-ubiquitinated CFTR was assessed by immunoprecipitation of CFTR and Western blotting for ubiquitin with the FK1 ubiquitin antibody in cells treated with ΔCif-OMV (Control) or Cif-containing OMV (90 min treatment). Western blotting with the FK1 ubiquitin antibody, that detects polyubiquitinated proteins, did not show a change in labeling after Cif treatment. Experiments were repeated at least 3 times.
(1.71 MB TIF)

Figure S2 G3BP1 knockdown, using additional target sequences, blocks the Cif-mediated degradation of CFTR. A. Transfection of two additional siRNA for G3BP1 reduces target protein abundance by 64% and 42% for construct #2 and #3, respectively. G3BP1 protein levels were determined by western blot analysis and quantification is present below a representative blot. B. siRNA for G3BP1 prevented the Cif-mediated lysosomal degradation of CFTR, as assessed by western blot analysis. Airway cells transfected with scrambled, siNeg control siRNA or siG3BP1 were treated for 60 minutes with ΔCif-OMV (Control) or Cif-containing OMV. Quantification for western blot experiments is presented below representative blots. Experiments were performed 3 times, * p<0.05 versus control.
(2.13 MB TIF)
