# Supplementary figures and images for: A Pseudomonas aeruginosa Toxin that Hijacks the Host Ubiquitin Proteolytic System
Source: PLoS Pathog. 2011 Mar 24;7(3):e1001325. doi: 10.1371/journal.ppat.1001325 (PMC3063759; doi:10.1371/journal.ppat.1001325)

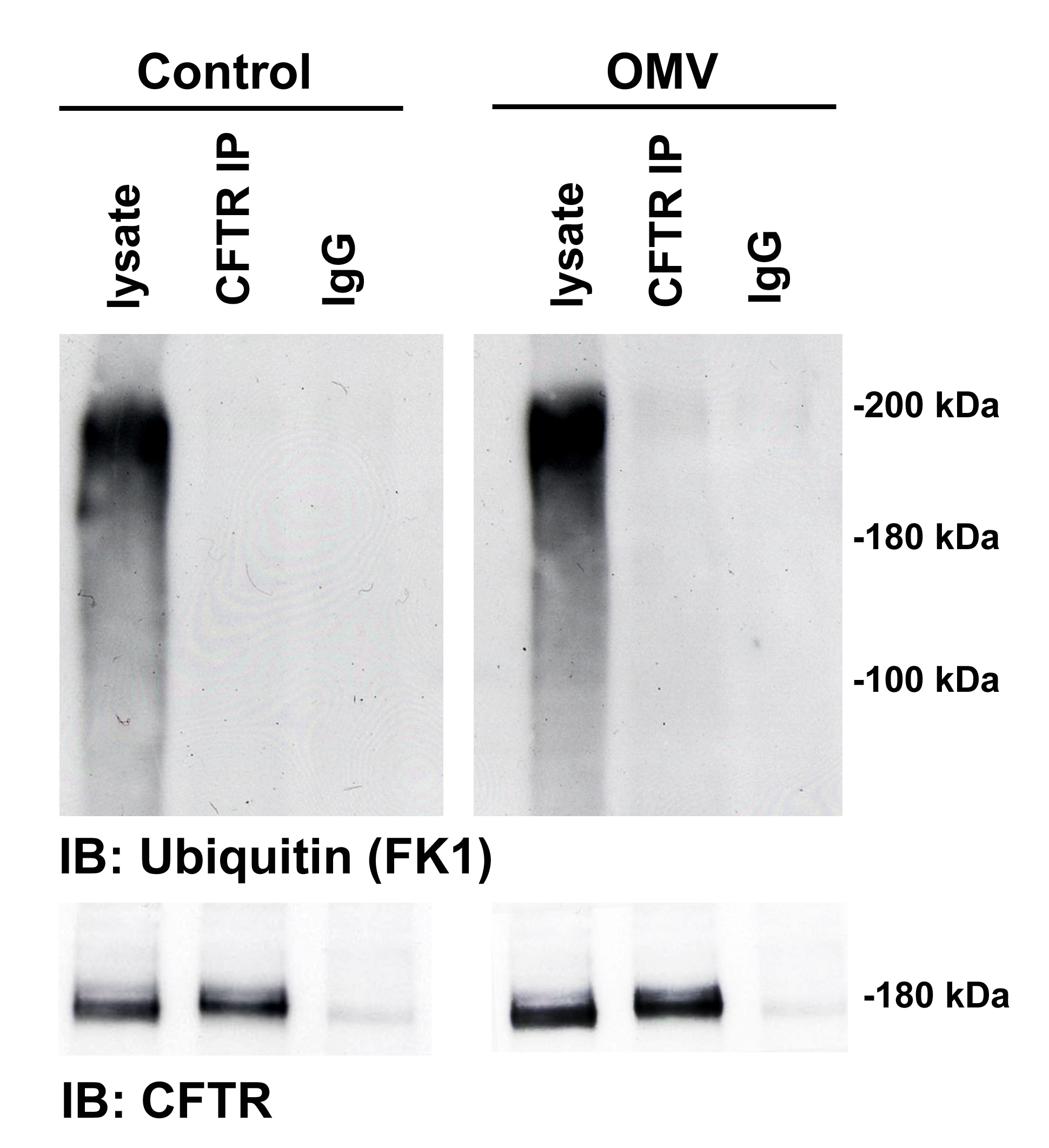

Supplement: Figure S1 — Cif does not alter the poly-ubiquitination of CFTR in polarized human airway epithelial cells. Poly-ubiquitinated CFTR was assessed by immunoprecipitation of CFTR and Western blotting for ubiquitin with the FK1 ubiquitin antibody in cells treated with ΔCif-OMV (Control) or Cif-containing OMV (90 min treatment). Western blotting with the FK1 ubiquitin antibody, that detects polyubiquitinated proteins, did not show a change in labeling after Cif treatment. Experiments were repeated at least 3 times. (1.71 MB TIF) [file ppat.1001325.s001.tif]

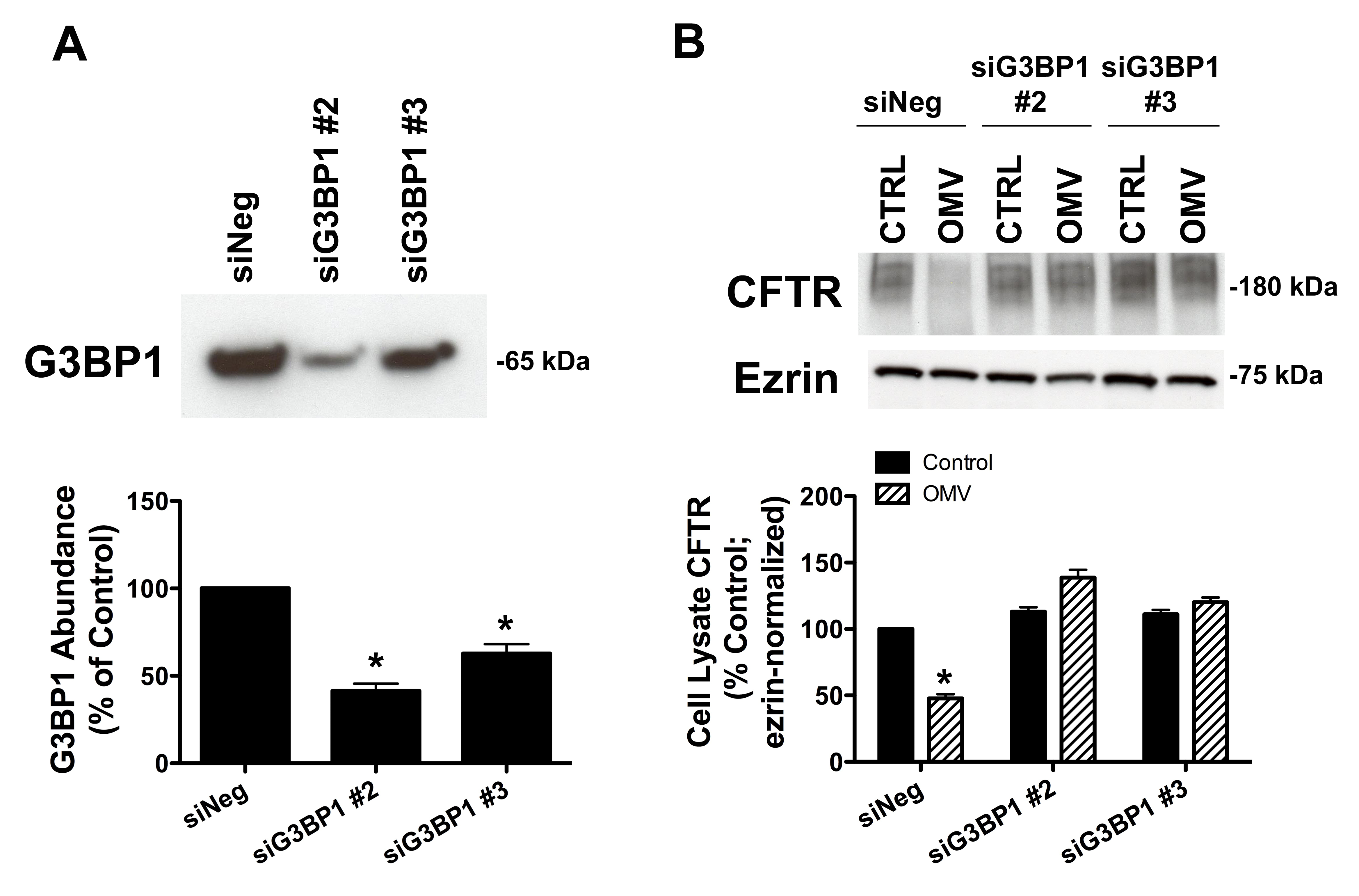

Supplement: Figure S2 — G3BP1 knockdown, using additional target sequences, blocks the Cif-mediated degradation of CFTR. A. Transfection of two additional siRNA for G3BP1 reduces target protein abundance by 64% and 42% for construct #2 and #3, respectively. G3BP1 protein levels were determined by western blot analysis and quantification is present below a representative blot. B. siRNA for G3BP1 prevented the Cif-mediated lysosomal degradation of CFTR, as assessed by western blot analysis. Airway cells transfected with scrambled, siNeg control siRNA or siG3BP1 were treated for 60 minutes with ΔCif-OMV (Control) or Cif-containing OMV. Quantification for western blot experiments is presented below representative blots. Experiments were performed 3 times, * p<0.05 versus control. (2.13 MB TIF) [file ppat.1001325.s002.tif]
